# Psychological Distress among Prostate Cancer Patients: Fact Or Fiction?

**DOI:** 10.4137/cmo.s955

**Published:** 2008-12-16

**Authors:** Christopher F. Sharpley, Vicki Bitsika, David H.R. Christie

**Affiliations:** 1 Centre for Bioactive Discovery in Health and Ageing, University of New England New South Wales, Australia; 2 Bond University, Queensland, Australia; 3 Premion, Queensland, Australia

**Keywords:** prostate cancer, psychosocial distress, depression, anxiety

## Abstract

Although the detrimental effect upon psychological well-being of receiving a diagnosis of, or treatment for, cancer has been demonstrated across many different types of cancer, three recent reviews of the psychological health of prostate cancer patients have produced contradictory conclusions. In order to elucidate the reasons for these apparent different conclusions, each of these reviews is described, with principal methods and findings summarised. Actual data, methodology used to select/reject research studies for inclusion in reviews, plus the validity of strict methodological culling of some research studies are discussed. Several extra studies and commentaries are also described, and a resolution of the apparent contradictory review conclusions is offered.

## Introduction

Receiving a diagnosis of cancer has been linked with a reduction in psychological well-being, Quality of Life, interpersonal relationships and optimism [1,2]. There are also data indicating that cancer patients who are anxious or depressed have poorer treatment outcomes, perhaps via lower compliance with medication [3], highlighting the need to address psychological functioning as an integral aspect of overall cancer treatment protocols. In support of this viewpoint, Greenberg [4: p. 1036] stated that “Inadequate treatment of major depressive disorders in this day and age is substandard oncology treatment”, a sentiment echoed in Australia by the NH&MRC *Clinical Practice Guidelines for the Psychosocial Care of Adults with Cancer* [5].

However, prior to administration of treatments aimed at alleviating the psychological sequelae of receiving a diagnosis of, and treatment for, cancer, the reliable identification of the incidence of psychological distress needs to be determined. Although this has been reported for several cancer patient groups (e.g. breast cancer [6]), there has been some debate as to the existence of elevated psychological distress among prostate cancer patients (PCa), with one review paper arguing that there was no real difference between the psychological distress of prostate cancer patients and their peers [7], but two other reviews claiming that the data from many studies indicate the presence of elevated anxiety and depression, plus lowered Quality of Life, among prostate cancer patients compared to their non-patient peers [8,9]. In order to resolve this apparent contradiction, the present paper examined the findings from those three major reviews of the previous research which investigated the presence of psychological distress among PCa patients. Each of these reviews will be described below, with some comments upon their methodologies, findings and conclusions. From that examination, overall findings regarding the presence (or not) of elevated psychological distress among prostate cancer patients will be discussed and the aforementioned disagreement between the three reviews will be resolved.

While there have been many research reports on the issue of psychological distress among prostate cancer patients, plus the three reviews mentioned above and described below, there has been no recent overall summary of the field to date, leaving the issue open to debate. Thus, this paper extends the previous literature by bringing together the previous major reviews and synthesizing their findings. The present structure and style of ‘reviewing the reviews’ was chosen instead of an alternate methodology (e.g. meta-analysis) because the process of gathering individual studies and reviewing them (as in the previous three reviews) has not produced a clear outcome to the questions of whether or not prostate cancer patients have higher levels of psychological distress than their peers. A further review of the original literature was considered not to be required at this juncture because performing another similar review might simply add to the disagreement shown by those three papers without advancing understanding of the overall literature to date and thus resolving the apparent disagreement between those previous reviews.

## Methods

The construct “psychological distress” was operationalised by reference to the nomenclature commonly used in the DSM-IV-TR [10] for “anxiety” and “depression” as representing the most common and researched indices of unhappiness of a psychological nature. In addition, and because they may be used as indicators of the relative absence of psychological distress, the global terms “psychological health” and “Quality of Life” were used to indicate the alternative “healthy” aspects of psychological status. “Quality of life” has been widely used in the health literature and may relate to a range of patient factors, including mental well-being, physical status and emotional health as assessed by the patient [11].

From a search of Pub Med, PsychInfo, Google Scholar to February 2008, using the descriptors “prostate cancer”, “depression”, “anxiety”, “psychological health” and “Quality of Life”, plus follow-up hand searches, three reviews of the literature were identified. Each of these three reviews was examined in terms of the articles they described, tabulations of data, measures of psychological distress used in the literature and conclusions drawn by the reviews. Each review was therefore “re-reviewed” by an examination of the original reports which had been reviewed and any discrepancies between the original data reported in the literature and the tabulations, descriptions or conclusions drawn by the three literature reviews were noted. On these bases, critical evaluations of the three literature were compiled and are presented below.

## Results

### Review 1: Bennet and Badger (2005)

Taken chronologically, Bennett and Badger [8] conducted the first comprehensive review of psychological distress among PCa patients from studies reported between 1988 and 2004. Those authors identified 38 studies (27 of which were descriptive and 11 that used experimental or quasi-experimental designs) from 39 published articles; 12 studies had samples of PCa patients that were less than 50 in number, eight studies reported on samples of between 50 and 100 PCA patients and 18 papers had samples of more than 100 PCa patients. Bennett and Badger focussed their review upon the incidence of depression among PCa men and noted that the Profile of Mood States was the most frequently used measure of depression, followed by the Hospital Anxiety and Depression Scale, the Beck Depression Inventory and the Center for Epidemiological Studies-Depression scale.

Bennett and Badger organised their review across five categories of depression incidence: those studies which reported on the prevalence and correlates of depression or other mood disorders (n = 9), papers that included depression in studies of fatigue and/or pain (4), studies of Quality of Life (QoL) and depression (12), the comparative incidence of depression among PCA patients and their partners (4), and intervention studies which used mood as the principal dependant variable (9). It is of note that, although Bennett and Badger criticised some studies on methodological grounds, they still included them in their review on the basis that these studies contributed valuable information to the discussion despite the presence of some methodological shortcomings. However, they did indicate that issues needing to be addressed were: lack of uniformity among measures used to tap depression, assessment of mood (via the POMS) rather than depression *per se*, low representation of some ethnic and social-cultural subgroups within the society where the largest number of research studies were conducted (U.S.A.); and that these shortcomings limited the generalisability of the overall conclusions from their review to the wider population of PCa patients. Bearing all these points in mind, Bennett and Badger nevertheless drew the conclusion that PCa patients experienced depressed mood at levels “higher than those reported for older men in the general population” (p. 554). Major risk factors for depressed mood were advanced PCa, prominent symptomatology and side effects from treatment, plus a prior history of depression. PCa patients in pain were also more likely to suffer depressed mood, whereas being married, receiving strong social support, being optimistic and having greater physical functioning were buffers against depressed mood in the samples studied. Of major import for the development of effective treatment options for PCa patients, Bennett and Badger noted that “the state of the science for supportive care interventions aimed toward men with prostate cancer is limited” (p. 554), thus emphasising the comments made by Greenberg (above) and the Guidelines of the NH&MRC and other similar national bodies that support provision of psychosocial care for all cancer patients as part of basic treatment protocols. This point is particularly relevant to the discussion of whether PCA patients actually do experience psychological distress at elevated frequencies compared to their non-PCa peers.

### Review 2: Katz (2007)

The second major review of PCa patients’ psychological well-being was reported by Katz [9], who reviewed studies of PCa and Quality of Life (QoL) from 1999 to 2005, a briefer period than that tapped by Bennett and Badger, but also including more recent data. Overall, Katz examined 68 reports of QoL among PCA patients, and specifically evaluated the effects of treatment modality (surgery, radiation therapy, cryosurgery) and also the effects of advanced PCa upon QoL. Katz’s conclusion mirrored that reported by Bennett and Badger: “Quality of Life is decreased in both the short and long terms for men with prostate cancer” (p. 302). However, one difference between the data reviewed by Bennett and Badger and that which Katz reviewed was the choice of the principal dependant variable. As mentioned above, Bennett and Badger included studies that used measures of mood (including depression) and QoL, whereas Katz restricted her review to QoL studies exclusively. Despite this different database, the conclusions drawn from these two reviews of PCa patients’ mood and QoL were congruent across these different dependant variables and thus form a significant statement regarding the psychological state of PCa patients.

It should, however, be noted that the use of QoL as an indicator of psychological distress may be flawed because QoL does not assess the accepted symptomatology of psychological maladjustment. Chen [12] commented that QoL measures are designed to assess the patient’s perception of the effects of symptoms upon daily role function and may/may not include the psychological impact of those symptoms. Thus, QoL measures are not designed to act as screening instruments for anxiety or depression and are not validated on agreed criteria for any disorder, leading to “much disagreement and confusion” about the exact meaning of QoL, with “researchers often using the same term (Quality of Life) to mean very different things” [12: p. 2695]. This means that the exact nature of QoL data is open to conjecture because of a “lack of standard criteria” [13, p. 626]. In terms of the implications of QoL data for intervention, Whelan et al. [14] noted that “Quality of life data are difficult for clinicians to interpret” because they do not relate to clinical symptoms (p. 6937). Thus, it could be argued that the value of QoL data in determining the psychological health of PCa patients (and their need for additional treatment) is limited. This does not invalidate Katz’s review but does restrict the generalisability of her findings to the specific issue of PCa patients’ psychological distress levels compared to their non-PCa counterparts.

By contrast, Nordin et al. [15] commented that, when investigating psychological distress and adjustment problems in cancer populations, “anxiety and depression are the most prevalent” indices to be assessed (p. 376). Both anxiety and depression are clearly defined in the ICD and DSM nomenclature and both are of major clinical significance to patients and patient care. Thus, the reviews by Bennett and Badger and Katz which respectively focused on depressed mood and QoL of PCa patients may be viewed as being limited in demonstrating that this patient group is similar to other cancer patients in experiencing increased levels of psychological distress While mood and QoL are of value when investigating the general mental states of participants, they are also somewhat distanced from the key diagnostic criteria of anxiety and depression [10] that constitute mental illnesses in themselves (and thereby represent a major treatment challenge for clinicians who deal with PCa patients). Therefore, it may be that measuring the presence of symptoms of anxiety and depression directly via ICD- or DSM-based instruments or procedures could be more informative than using patients’ reflections upon their mood or level of functioning and how these make them feel. On this basis, the focusing of research reviews upon those studies which apply valid and reliable measures of anxiety and depression to PCa patients is probably necessary to adequately address the issue of PCa patients’ psychological distress.

### Review 3: Bloch et al. (2007)

The third major review of PCa patients’ psychological distress is also of value because it adopted a different approach to literature selection from that followed by Bennett and Badger and Katz. Bloch et al. [7] examined studies on the psychological effects of PCa that were reported between 1988 and June, 2006, noting that that “Few of the … investigations have enlisted large samples or controls” (p. 3), leading those authors to exclude studies that were “methodologically inadequate” (p. 545). Applying these criteria, Bloch et al. found just three methodologically acceptable cross-sectional studies, plus two longitudinal studies of PCa patients’ psychological distress and concluded from these five studies that there were “few differences in levels of psychological distress” (p. 11) between men with and without PCa on the basis of “studies with reasonably sized and unbiased samples with appropriate designs, including adequate descriptions of instruments used” (p. 2). Because of its relatively different conclusions to those from Bennett and Badger and Katz, it is worth examining the procedures and findings of the Bloch et al. review in some greater detail to determine the validity of those conclusions vis-à-vis those of Bennett and Badger and Katz (even though those reviews were not specifically focussed upon psychological distress as defined in the DSM or ICD terms). As a first step in that process, it is of relevance to examine the three cross-sectional and two longitudinal studies upon which Bloch et al. based their conclusion (Bacon et al. [16], Clark et al. [17], Helgason et al. [18], Nordin et al. [15] and Visser et al. [19]).

Bacon et al. [15] reported that PCa patients had significantly worse scores on 7 out of 10 QoL subscales than controls but not on the mental subscale. Although the samples were restricted to a particular occupational group (i.e. health professionals) and thus results are limited in their generalisability to the population of PCa patients, data do indicate that these PCa patients were experiencing a significantly lower overall quality of life than non-PCa men. Similarly, the widespread negative attitudes reported by PCa patients in Clark et al.’s [17] study were highlighted by those authors, who also commented on the unreliability of focussing upon the lack of differences between PCa patients and controls in global measures of health status. Helgason et al. [18] described their PCa sample as “significantly more likely to be distressed” (p. 1418) than non-PCa men due to decreases in sexual functioning. These three studies constituted what Bloch et al. described as those which “stand out since the samples are adequate and compared with controls” (p. 2), but they do not support Bloch et al.’s conclusion that “Men with PCa do not appear to experience marked impairment of adjustment” (p. 12). In addition, none of the three cross-sectional studies accepted by Bloch et al. as methodologically satisfactory actually used valid and reliable measures of depression or anxiety. Instead, these three papers used QoL, patient “attitudes” and “distress” as their DVs. While these data are valuable in describing the emotional states of the PCa patients examined, they do not assess anxiety or depression as defined in ICD or DSM terms.

Of the two longitudinal studies of psychological adjustment among PCa patients that met Bloch et al.’s methodological criteria for inclusion, Nordin et al. [15], collected HADS data from 118 PCa patients close in time to their diagnosis and then again from 99 of these patients six months later. Data were also collected from breast, colorectal and gastric cancer patients. Unfortunately, the HADS data reported were the group means for PCa and other patient groups rather than the percent who reached the cutoff score indicating an anxiety or depression disorder. Thus, it is not possible to determine the incidence of clinically depressed or anxious PCa patients at either diagnosis or six months later. Visser et al. [19], used a QoL instrument as well as a shortened version of the Profile of Mood States (POMS). Although the shorter POMS has a depression subscale, scores on the latter were not reported.

Thus, the data from the three cross-sectional and two longitudinal studies referred to by Bloch et al. as indicating a lack of difference in the psychological distress of PCA patients versus their non-PC a peers may be of limited value because four of those studies used QoL as the main DV rather than measures of clinical anxiety or depression and also because the single study which did use a standardised measure of anxiety and depression (the HADS) did not report incidence data for the PCa sample which reached clinical levels of anxiety or depression. In addition, there were, in fact, several key datum which indicated that PCa patients were less happy with their health than non-PCa peers.

By contrast, of the three cross-sectional papers that focussed upon depressive symptoms and which were rejected by Bloch et al. on the basis of methodological weaknesses (Heim and Oei [20], Stone et al. [21], Balderson and Towell [22]), Heim and Oei [20] reported that 32% of their sample of PCa patients met the criteria for a clinical diagnosis of depression on the BDI, many times more than the 3% or less reported in national surveys of the older male population in Australia, where Heim and Oei collected their data. Stone et al.’s [21] data were not directly relevant to this discussion because PCa patients’ combined anxiety-depression mean scores (from the HADS) were not reported separately to other cancer patient groups, but Balderson and Towell [22] found 38% of their PCa sample met the criteria for combined anxiety-depression on the HADS, well above the norms for this age group.

## Discussion

These three reviews demonstrate some uncertainty on the issue of PCa patients’ psychological distress. As well as reporting two different conclusions, each of these reviews adopted a different stand on selection of studies to be reviewed. Bennett and Badger [8] included all 39 studies of depression among PCa patients that they identified via electronic search and concluded that, while PCa patients were, on balance, more distressed than their non-PCa peers, the predominant measures used were focussed upon mood rather than ICD- or DSM-based anxiety or depression; Katz [9] restricted her review to those studies which used QoL as the principal dependant variable rather than psychological disorders, again including all studies regardless of methodological weaknesses and also concluding that PCa patients had relatively poorer QoL than non-PCa men. By contrast, Bloch et al. [7] excluded studies on the basis of strict methodological criteria and concluded (on the basis of those few studies which remained after exclusion) that there were no significant differences in the psychological distress levels of PCa patients and their non-PCa peers.

As well as presenting contradictory conclusions, the three reviews described above raise several issues for further examination, principally those of: choice of DV and measures, selection of control groups or reference to normative data for purposes of comparison, the effects of research design variables upon validity of data reported and sample size. While the soundness of research methodology is undeniably an important aspect of selection of which studies to include in literature reviews, those methodological criteria need to be carefully considered before implementation. Specifically, although (as argued by Bloch et al.) cross-sectional ‘snapshots’ do not provide data on the persistence of symptoms, they do tell us if there is a discrepancy between the scores of a certain sample on a selected variable at a point in time when they are compared to another group or set of norms. While it would be valuable to know the features of depression and anxiety over time among PCa patients, the lack of such longitudinal knowledge does not preclude clinicians recognising that such distress exists and that it can hinder treatment and be detrimental to patient health and well-being. So, although a temporal “map” of psychosocial distress across all periods of PCa diagnosis, treatment and recovery is clearly of value (and such data have been reported [23,24]), simple snapshots are not worthless—they indicate whether this population *ever* experiences psychosocial distress that warrants intervention.

A major methodological aspect of cross-sectional and longitudinal studies is the presence of control groups, which Bloch et al. described as “crucial” (p. 12). However, this is not necessarily the case in research which involves psychometric testing, where the accepted procedure is to refer to established norms when comparing the scores of a selected sample. For example, data from the Australian National Survey of Mental Health and Well-Being [25] showed that anxiety and depression decline with age [26], and that the prevalence of anxiety and depression among men aged 55 to 64 was 6% and 3% respectively, dropping to 3.5% and <1.0% after age 65 [27]. Data collected from a sample of Australian PCa patients (at any time after their diagnosis) may be profitably compared with these norms when drawing conclusions about the effects of PCa upon patients, thus obviating the need for another ‘control’ group. Similarly, the issue of adequate sample size is open to some further interpretation than simply classifying studies as methodologically insufficient because they use samples that are smaller than might be ideal. Power analysis may be profitable where the null hypothesis has been accepted, but *ad hoc* rejection of studies simply on the basis of sample size is probably overly-hasty. It was pointed out some time ago by Glass, McGaw and Smith [28] that the sample size of a particular study can be coded and evaluated for consideration when reviewing the overall findings of literature reviews.

However, the major issue that arises from these three reviews is the choice of dependant variables used to assess the incidence of psychological distress. There is a clear difference between QoL and a psychiatric diagnosis of anxiety or depression and there are different issues being investigated when QoL is the DV than when ICD- or DSM-based diagnoses of anxiety and depression are made. We would agree with Nordin et al. [15] that it is the latter which command the first attention of researchers who investigate the psychological effects of PCa upon patients and the consequent need for intervention to address those mental health problems which may interfere with treatment compliance and adversely affect patients and their relationships.

Although on a purely numerical basis (i.e. 2 out of 3), the overall conclusion that could be drawn from the above evaluation of the three literature reviews would that there is evidence of elevated levels of unhappiness or depressed mood (including QoL) among PCa patients compared to their non-PCA peers, this is not clear-cut, leaving the question open to some conjecture. While conducting another comprehensive literature review so soon after these three might bear relatively poor cost-benefit outcomes, an alternative way to further examine these findings is to set up a test of the null hypothesis (i.e. that there is no significantly different level of psychological distress among PCa patients compared to non-PCa men of similar age). This test may be conducted by randomly selecting a discrete number of studies which examined the research question using sound methodology and valid and reliable instrumentation. Although it is impossible to determine the exact number of such studies needed to conclusively and finally reject the null hypothesis in question herein, the advice of Kazdin [29] that systematic replication (i.e. “repetition of the experiment by systematically allowing features to vary” (p. 489)) may be followed by selecting studies which showed some variability in terms of national origin of sample, instruments used, settings where data were collected and time collected (i.e. from initial biopsy to post-treatment).

### Resolving the contradiction: Testing the null hypothesis

On this basis, we gathered a small random selection of recent studies to act as a vehicle for testing the claim that there are no significant differences between the levels of psychological distress of PCa patients and their non-PCa peers. These studies are presented as a representative sample of the wider recent literature but are not intended to constitute a comprehensive review. Rather, they are described to offer a brief indicator of the kind of research into the psychological distress experienced by PCa patients that has been published recently and, as such, to pose a test of the null hypothesis that there is no significant difference between the level of psychological distress of PCA patients and their non-PCA peers.

From a Google Scholar search in March, 2008, with the descriptors “prostate cancer, anxiety, depression” for the period 1997 to 2007, we identified six studies which reported upon the incidence of clinically significant anxiety and/or depression among PCa patients. As well as the criteria for systematic replication mentioned above, these studies were selected on the bases of (a) having used well-recognised measures of anxiety and/or depression, (b) having sufficiently large samples to allow generalisability to the population, (c) being from national sources where norms of the incidence of anxiety and depression were available in some form, and (d) using a recognized research design that was generally free from major sources of experimental invalidity. To adequately represent the national source of most studies of PCA, four studies that collected data from men in the U.S.A. were identified. Second, to provide a cross-cultural comparison, one study from a similar society (Australia) and one study from a dissimilar society (Japan) were also selected. Each of these six studies is briefly described below, followed by a summary of these data and their implications for consideration of the incidence of psychological distress among PCa patients.

## Studies selected

Dale, Hemmerrich and Meltzer [30] assessed anxiety among 178 prostate cancer patients with an average age of 63.1 years who were recruited at the Chicago University at the time of biopsy (i.e. prior to treatment but with an awareness that PCa might become their formal diagnosis). Those authors reported that the mean HADS-A score was 3.70 (with 7 being the cutoff for presence of clinically significant anxiety) but that 18% of their sample had scores above this cutoff level, indicating the presence of “at least mild clinical anxiety” (p. 496).

Pirl, Gebrielle, Siegel, Goode and Smith [31] examined the results of Structured Clinical Interviews for DSM-IV (SCID) and BDI scores of 45 men aged between 51 and 83 years (M = 69.4 years) who had been diagnosed with PCa for an average of 6.4 years and who were patients at the Massachusetts General Hospital. According to the SCID, 12.8% of the sample qualified for “major depressive disorder” (p. 521), and 13.3% of the sample met the criteria for mild to moderate depression on the BDI.

Monahan, Champion, Rawl, Giesler, Given, Given et al. [32] assessed depression among 105 PCa patients aged from 42 to 80 years (M = 64 years) who were “newly treated” (p. 401) for PCa via radical prostatectomy, radiation therapy or brachy-therapy in Indiana, Michigan and Louisville. The instrument used to assess depression was the Center for Epidemiologic Studies-Depression Scale (CES-D), which identified 16% of the sample as having clinical depression.

Roth, Kornblith, Batel-Copel, Peabody, Scher and Holland [33] used the HADS to assess anxiety and depression in 121 PCa patients from New York who had an average age of 71 years (range = 52 to 88 years) and who had received their PCa diagnosis a median of 4 years previously. Thirty-two percent of the sample scored above the HADS anxiety cutoff and 15.2% scored above the HADS depression cutoff. In addition, 17 of these patients were assessed by a psychiatrist and eight met the criteria for a DSM-IV disorder.

Sharpley and Christie [34] assessed anxiety and depression among 185 Australian PCa patients aged from 54 to 81 years (M = 69.2 years) who had received their diagnosis an average of 1 year, 10 months previously. Patients were asked to complete the Zung Self-Rating Anxiety Scale (SAS) and the Zung Self-Rating Depression Scale (SDS). The incidence of clinical anxiety disorder and depressive disorder on the SAS and SDS were 11.8% and 13.3% respectively.

In keeping with Kazdin’s [29] recommendations regarding systematic replication, the data reported in these five papers were collected from men from the U.S.A. or Australia, two similar societies and nations for which data are available regarding the national incidence of anxiety and depression. One study assessed anxiety alone, two examined depression alone and two collected data on both anxiety and depression. In addition, several different instruments and procedures were used to collect these data, including the HADS, BDI, DSM-IV Structured Interviews, CES-D, and Zung’s SAS and SDS, all of which possess satisfactory validity and reliability. With samples of between 45 and 185 PCa patients, none of these reports could reasonably be described as so small as to invalidate the data reported therein. Finally, data were collected from immediately before formal diagnosis [30], soon after diagnosis [32] and at two, four and six years after diagnosis [34,33,31]. Thus, if there were consistent findings across (a) nations, (b) samples within nations, (c) instruments and procedures and (d) time after (or before) diagnosis, the requirements of systematic replication might be seen to have been observed. Further, if these findings were indicative of levels of anxiety and depression that were elevated above the reported national norms, then it might be reasonable to draw some conclusions regarding the effects of PCa upon patients’ psychological well-being among the wider population.

By reference to the DSM-IV-TR [10], the general community 1-year prevalence rate for Generalised Anxiety Disorder is about 3% and the point prevalence rate for Major Depressive Disorder in between 2% and 3% for men. While these data apply to the whole of lifespan, Form [35] reviewed 27 studies of age differences in depression, 10 of the prevalence of anxiety disorders and 13 of distress and concluded that “age is associated with less anxiety and depression” (p. 20). These APA [10] rates are most relevant to the U.S.A. but figures from Australia [26,27] mentioned earlier in this paper state that the prevalence of anxiety is between 6% and 3.5% for men aged 55+, and for depression the prevalence rate is between 3% and <1%. Using these norms for the two relevant populations (i.e. U.S.A., Australia) obviates the need for control groups and shows that the rates reported in the five studies mentioned above are well above those that might be expected for comparably-aged men in the U.S.A. or Australia. While the direct comparisons with the APA data are only able to be made where the DSM-IV-Structured Interview process has been used (i.e. as by Pirl, Gebrielle, Siegel, Goode and Smith [31]), those data indicated that 12.8% of their sample met the criteria for Major Depressive Disorder, more than four times that which might be expected. The data collected on Australian men with PCa [34] indicated that 11.8% were clinically anxious, nearly double the population rate of 6%; the incidence of depression (16%) in that sample was over five times the 3% rate for depression expected from people of the same age range in Australia [26]. Thus, there is consistency of outcome across the data from U.S.A. and Australia, plus consistency across those studies conducted only with U.S.A. participants, thus providing some within-population replication. Further, although the instruments and procedures varied, the findings are generally in agreement across them. That is, although the BDI focuses upon mostly cognitive symptoms, the HADS excludes those physical symptoms which may be associated with disease, the SAS and SDS include items that tap the range of symptoms which comprise DSM-based criteria, and the SCID directly assesses the presence of those DSM criteria, the levels of anxiety and depression encountered within these samples and with these instruments are uniform in being elevated well above the norms for the age groups sampled. Finally, there is consistency in the finding of elevated anxiety and depression among PCa samples regardless of how soon they were assessed after (or before) formal diagnosis. Thus, it appears reasonable to conclude that there is sufficient evidence of anxiety and depression among PCa patients in these five studies to conclude that they experience elevated psychological distress compared to their non-PCa peers.

As mentioned above, all of these five studies were from Western populations and so a further study conducted on Japanese men was also examined. By adding “Japan” to the search criteria described above (i.e. “prostate cancer, anxiety, depression”) the report by Namiki, Saito, Tochigi, Numata, Ioritani and Arai [36] was located. Those authors investigated the presence of psychological distress among 340 Japanese PCa patients and used some QoL measures and the HADS-A and HADS-D, plus a combined measure of these two subscales called HADS-T. Patients were classified as anxiety or depression “cases” based upon a previous study of Japanese PCa patients’ normative data [37]. On this basis, 114 (33.5%) of the sample were found to be anxiety and depression “cases.” Although national norms of the incidence of anxiety and depression in Japan were not obtained, it is highly unlikely that they would show a third of men in the age group studied to be clinically depressed or anxious. Thus, these cross-cultural data reinforce the conclusions drawn from the USA and Australian data reported in the previous five recent studies and provide a systematic replication of the examination of the levels of psychological distress among PCa patients compared with non-PCa peers. While there is no upper limit to the number of systematic replications needed to provide complete generalisability [38], the consistent findings reported by all six studies across settings, nationalities, societies, instruments, stage of PCa development and treatment, present substantial evidence to refute the null hypothesis that there are no significant differences between the levels of psychological distress of PCa patients compared to their non-PCA peers.

## Commentaries

In addition to these six reports on anxiety and depression among PCa patients, five other recent papers were briefly considered. Mehnert, Lehmann, Schulte and Koch [39] found that 53% of 197 PCa patients experienced “prevalent” nervousness, worries, fears and sadness. Although these authors did not report formal DSM-IV-TR disorders, they raised the important point that many (in this case over half) of their sample of PCa patients were significantly psychologically distressed by their diagnosis and treatment. When added to Sellick and Crooks’ [40] findings that 19% to 43% of cancer patients experienced high levels of depressive symptoms and 6% to 15% met the strict criteria for a clinical diagnosis of major depression; that depression has been shown to adversely affect treatment efficacy [41]); and that the major unmet need reported by cancer patients is in regard to their psychological distress [42], it appears clear that at least some PCa patients (many more than would be expected among their non-PCa peers) suffer psychological distress worthy of clinical attention and treatment, just as do all other cancer patient groups so far investigated.

Although there were no conclusive data presented by any of the three reviews which supported the null hypothesis, it is worthwhile to note Katz’s [9] comment that, in regard to the stress of PCa diagnosis and treatment, “Men may not complain about these symptoms when interacting with healthcare professionals” because they wish to focus upon a “ ‘survival at all costs’ attitude, and they may accept any and all side effects without complaint.” (p. 306). This comment is worthy of consideration because it points to a potentially confounding variable in studies of PCa patients’ psychological distress that may lead to Type II errors when evaluating the incidence of clinical distress among this patient group.

## Conclusion

There is no doubt that attention to issues of methodology, research design and some of the other criteria commonly used to asses the validity of research studies can promote scientific reflection. However, there are sufficient data reported so far, plus clinical observations aplenty, to assume that receiving a diagnosis or treatment for PCa is highly likely to be a significantly distressing occasion for a substantial proportion of these men. It appears from the data reviewed herein (both from the previous reviews and from the later selection of six studies) that PCa patients experience levels of anxiety and depression that are elevated above those of their fellows, with a greater incidence of clinically significant anxiety and depression overall than men without PCa. Just as for other cancer patients, this is a most challenging and sometimes psychologically distressing experience for many men and (also as for other cancer patients), receiving such a diagnosis may lead to anxiety and depression which adversely influence these men’s relationships with others [1,2] and adherence to treatment [3], thus further complicating the provision of treatment and potentially reducing its effectiveness.

Although not the primary focus of the present paper, some comment may be made regarding recommendations for clinicians when they are delivering the diagnostic information which has been shown above to lead to psychological distress. First, there are some data which suggest that anxiety and depression among cancer patients may be minimized by adequate explanations of diagnostic information and treatment options [43,44]. Other data indicate that an individually-focussed approach to each patient’s informational needs, which might include a tour of the treatment facility or online access to the patient’s own records, can lead to reduced psychological distress [45]. Second, it must be accepted that receiving a diagnosis of cancer will be distressing for most people and therefore this reaction should be seen as part of the overall process which will, according to previous studies, reduce over time [46]. While they may (and should) be aware of the particular informational needs of their patients, including the fact that a few patients will prefer not to receive any information at all [47], clinicians need not set a goal of a distress-free consultation when delivering a diagnosis of prostate cancer. As has been argued at length elsewhere [48,49], the ‘normal’ response to experiencing aversive events is to withdraw, prepare for the worst outcome and reflect upon mortality, and this is most likely to occur to many prostate cancer patients. The provision of supportive psychological therapies which focus upon the therapist-patient rapport [50] by associated health staff, plus monitoring of patient emotional well-being may be effective avenues of clinician response to the psychological distress which they are likely to encounter in their prostate cancer patients.
